# Bibliometric-analysis visualization and review of non-invasive methods for monitoring and managing the portal hypertension

**DOI:** 10.3389/fmed.2022.960316

**Published:** 2022-09-15

**Authors:** XiaoHan Sun, Hong Bo Ni, Jian Xue, Shuai Wang, Afaf Aljbri, Liuchun Wang, Tian Hang Ren, Xiao Li, Meng Niu

**Affiliations:** ^1^Department of Interventional Radiology, The First Hospital of China Medical University, Shenyang, China; ^2^Department of Interventional Therapy, National Cancer Center/National Clinical Research Center for Cancer/Cancer Hospital, Chinese Academy of Medical Sciences and Peking Union Medical College, Beijing, China

**Keywords:** portal hypertention, bibliometric, elastography, non-invasive, VOSviewer

## Abstract

**Background:**

Portal hypertension monitoring is important throughout the natural course of cirrhosis. Hepatic venous pressure gradient (HVPG), regarded as the golden standard, is limited by invasiveness and technical difficulties. Portal hypertension is increasingly being assessed non-invasively, and hematological indices, imaging data, and statistical or computational models are studied to surrogate HVPG. This paper discusses the existing non-invasive methods based on measurement principles and reviews the methodological developments in the last 20 years.

**Methods:**

First, we used VOSviewer to learn the architecture of this field. The publications about the non-invasive assessment of portal hypertension were retrieved from the Web of Science Core Collection (WoSCC). VOSviewer 1.6.17.0 was used to analyze and visualize these publications, including the annual trend, the study hotspots, the significant articles, authors, journals, and organizations in this field. Next, according to the cluster analysis result of the keywords, we further retrieved and classified the related studies to discuss.

**Results:**

A total of 1,088 articles or review articles about our topic were retrieved from WoSCC. From 2000 to 2022, the number of publications is generally growing. “World Journal of Gastroenterology” published the most articles (*n* = 43), while “Journal of Hepatology” had the highest citations. “Liver fibrosis” published in 2005 was the most influential manuscript. Among the 20,558 cited references of 1,088 retrieved manuscripts, the most cited was a study on liver stiffness measurement from 2007. The highest-yielding country was the United States, followed by China and Italy. “Berzigotti, Annalisa” was the most prolific author and had the most cooperation partners. Four study directions emerged from the keyword clustering: (1) the evaluation based on fibrosis; (2) the evaluation based on hemodynamic factors; (3) the evaluation through elastography; and (4) the evaluation of variceal bleeding.

**Conclusion:**

The non-invasive assessment of portal hypertension is mainly based on two principles: fibrosis and hemodynamics. Liver fibrosis is the major initiator of cirrhotic PH, while hemodynamic factors reflect secondary alteration of splanchnic blood flow. Blood tests, US (including DUS and CEUS), CT, and magnetic resonance imaging (MRI) support the non-invasive assessment of PH by providing both hemodynamic and fibrotic information. Elastography, mainly USE, is the most important method of PH monitoring.

## Introduction

Portal hypertension (PH), defined as portal pressure gradient (PPG) or hepatic venous pressure gradient (HVPG) > 5 mmHg, nearly 90% is cirrhotic PH, which belongs to intrahepatic PH (1). Cirrhotic PH is the result of the growing hepatic sinusoidal resistance and splanchnic blood flow. Cirrhosis can be caused by various factors like viruses, alcohol, drug, and fat, which lead to inflammatory necrosis and fibrosis of hepatocytes. The nodular regeneration surrounded by a dense fibrous septum and the dedifferentiation of capillary-like hepatic sinusoids induced by chronic inflammation led to the distortion and the high resistance of intrahepatic vessels (2, 3). In addition to the structural changes, chronic hepatitis increases the intrahepatic vascular tone by reducing the activity of vasodilative factors like NO (4). When portal vein blood could not flow smoothly through the liver to the inferior vena cava, the portal pressure rises and stimulates the secretion of vasodilator in the splanchnic blood vessels and the formation of collateral circulation, neovascularization, and hyperdynamic state. In turn, it exacerbates the hyperemia of splanchnic circulation and portal hypertension (5). Overall, the intrahepatic factors included collage deposition and the imbalance of vasodilation and vasoconstriction, while the extrahepatic factor was hyperdynamic circulation.

HVPG, the golden criterion to assess the presence and severity of portal hypertension, is the difference between wedge-shaped hepatic venous pressure (WHVP) and free hepatic venous pressure (FHVP) (6). Grading of portal hypertension in cirrhosis helps to stratify patients with a poorer clinical course: HVPG > 5 mmHg is associated with chronic hepatitis progression and hepatitis recurrence after liver transplantation (7, 8); HPVG > 10 mmHg represents clinically significant portal hypertension (CSPH), predicts the development of varices and ascites, and high risk of decompensation and recurrence of liver cancer after hepatectomy (9–13); HPVG > 12 mmHg significantly increases the risk of variceal bleeding (14); when HPVG > 16 mmHg, the risk of death was significantly increased (15). For cirrhotic patients awaiting liver transplantation, for every 1 mmHg increase in HVPG, the risk of death is increased by 3% (16). Therefore, as outlined in the major clinical practice guidelines (17–20), the routine measurement of portal vein pressure is of great significance for the management of the whole course of liver cirrhosis, not only as an index to evaluate the prognosis but also as an important basis to determine the direction of treatment.

The high technical difficulty, high cost, invasiveness, and low feasibility of multiple measurements restrict the widespread clinic application of HVPG (21). Otherwise, one study (22) in cirrhotic patients with non-alcoholic steatohepatitis (NASH) shows that the consistency between WHVP and portal pressure (PP) is poorer than in cirrhotic patients with alcohol- or virus-related hepatitis, and HVPG may underrate the level of PH in the NASH-related cirrhosis. Although the studies (23, 24) about endoscopic ultrasound (EUS)-guided PPG measurement have proven its accuracy compared to HVPG, which could directly obtain PP with fewer injuries, EUS-guided PPG measurement is still a high-risk operation with a great requirement of equipment and technology. Hence, the non-invasive detection of portal hypertension is necessary and urgent. Based on the developing mechanisms of portal hypertension, the indices of liver fibrosis, liver function, intrahepatic endothelial dysfunction, inflammation, and intra- or extra-hepatic hemodynamics have been assessed to substitute HVPG. Moreover, the rapid rise of radiomics and artificial intelligence (AI) revolutionarily changes the diagnosis and management of liver disease, which can fully exploit the uses of the information from routine imaging and serological examinations. The increasing studies successfully estimate the portal pressure by constructing a computer model with superior performance.

This article reviews the development of non-invasive detection of portal pressure with the assistance of bibliometric-analysis visualization and analyzes the current shortcomings and future expectations, in hopes of the routine monitoring of PH being a reality in the clinic.

## Methods

**FIGURE 1:**
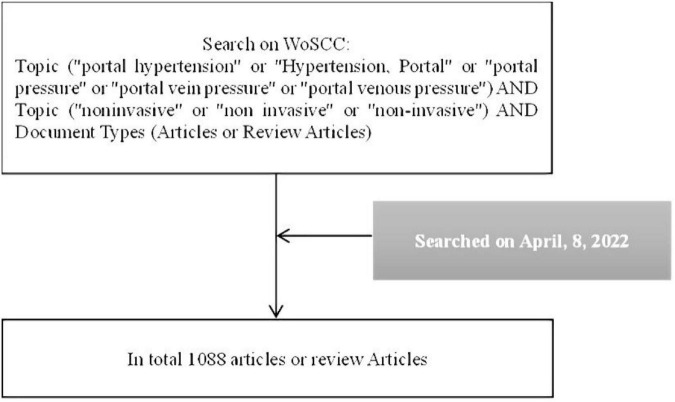
Shows the main processes of data collection and screening and analysis.

On 8 April 2022, we retrieved a total of 1,209 records (publication time from 2000 to 2022) on the Web of Science Core Collection (WoSCC) by searching the term Topic (“portal hypertension” or “Hypertension, Portal” or “portal pressure” or “portal vein pressure” or “portal venous pressure”) AND Topic (“non-invasive” or “non-invasive” or “non-invasive”). A total of 121 meeting abstracts, editorial materials, letters, corrections, and reprints were excluded. Approximately, 1,088 articles or review articles were included in this study. The full record and cited references of the 1,088 articles were exported in the format of a plain text file. Figure 1 shows the process of data collection.

Next, the bibliographic data were imported to VOSviewer 1.6.17.0 to create analytic maps. VOSviewer was a feature-rich bibliometric analysis tool, and the abundant bibliometric information in WOS data made it play completely. The analytic results were saved in the format of a figure and were exported to Microsoft Office Excel 2021. The analysis of the trend of annual publications and the rank of authors, regions, organizations, journals, literature, and keywords were finished in Excel. Based on keyword clustering, the four clusters inspired further discussion of non-invasive methods for PH detection.

## Results

### Annual publication

**FIGURE 2:**
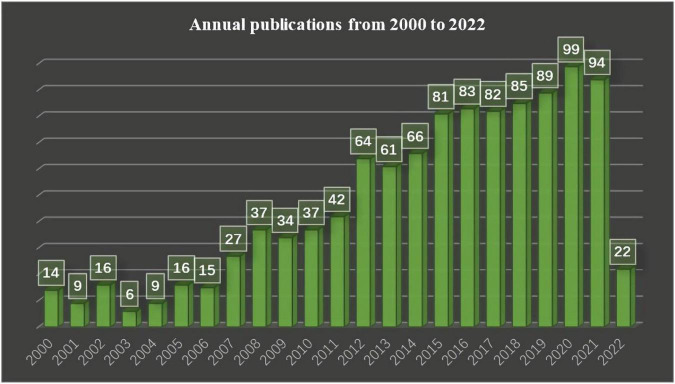
Shows the number of articles published in different years in the field of non-invasive prediction of portal hypertension over a period of 22 years.

Figure 2 shows that the number of published related articles is increasing year by year. It started to rise rapidly in 2007, and, since 2012, the number of published related articles has stabilized at more than 60. At present, 2020 is the year with the most published articles, with 99 articles in total.

### Top 10 high-yielding journals

There were in total 306 journals of 1,088 searched pieces of literature, and the top 10 high-yielding journals are listed in Table 1, the 10 journals published the 25% retrieved literature, of which, “Journal of Hepatology” and “Hepatology” were the top 2 high-cited journals with citations of 4,920 and 2,663, respectively, while “World Journal of Gastroenterology” published the most articles.

**TABLE 1:** Top 10 high-yielding journals.

Rank	Journal	Documents	Account for	Citations	IF
1	World Journal of Gastroenterology	43	4%	811	5.742
2	Journal of Hepatology	39	4%	4,920	25.083
3	Liver International	31	3%	814	5.828
4	Hepatology	28	3%	2,663	17.425
5	European Journal of Gastroenterology and Hepatology	25	2%	363	2.566
6	Digestive and Liver Disease	23	2%	611	4.088
7	Ultrasound in Medicine and Biology	22	2%	763	2.998
8	Hepatology Research	21	2%	203	4.288
9	Journal of Ultrasound in Medicine	21	2%	315	2.153
10	Journal of Gastroenterology and Hepatology	20	2%	484	4.029
			25%		

### Top 10 high-cited documents

There were in total retrieved 1,088 documents, and the 10 documents in Table 2 were the highest cited. Among them, the 4th and 7th were practice guidelines about the application of ultrasound elastography and the non-invasive measurements of liver disease; the 1st, 2nd, 3rd, 6th, and 9th were reviews about liver disease and non-invasive measurements of liver disease; the 5th, 8th, and 10th were articles about liver stiffness measured by transient elastography (TE) and magnetic resonance elastography (MRE), and platelet count/spleen diameter in screening esophageal varices, respectively. Of the 10 publications, 3 were published by “Journal of Hepatology.”

**TABLE 2:** Top 10 high-cited documents.

Rank	Title	Journal	Citations	Pub. year	References
1	Liver fibrosis	Journal of clinical investigation	3,605	2005	(135)
2	Liver cirrhosis	Lancet	1,286	2008	(2)
3	Non-invasive evaluation of liver fibrosis using transient elastography	Journal of Hepatology	939	2008	(136)
4	EASL-ALEH clinical practice guidelines: non-invasive tests for evaluation of liver disease severity and prognosis	Journal of Hepatology	861	2015	(137)
5	Liver stiffness measurement predicts severe portal hypertension in patients with HCV-related cirrhosis	Hepatology	495	2007	(103)
6	Non-invasive methods to assess liver disease in patients with hepatitis b or c	Gastroenterology	419	2012	(138)
7	WFUMB guidelines and recommendations for clinical use of ultrasound elastography: part 3: liver	Ultrasound in medicine and biology	393	2015	(139)
8	Magnetic resonance imaging more accurately classifies steatosis and fibrosis in patients with non-alcoholic fatty liver disease than transient elastography	Gastroenterology	381	2016	(140)
9	Complications of cirrhosis. I. Portal hypertension	Journal of Hepatology	373	2000	(141)
10	Platelet count/spleen diameter ratio: proposal and validation of a non-invasive parameter to predict the presence of esophageal varices in patients with liver cirrhosis	Gut	313	2003	(128)

### Co-authorship of countries and regions/organizations

**FIGURE 3:**
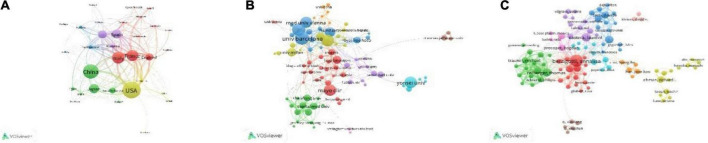
Shows the research level and connections of different countries and regions, institutions, and researchers. **(A)** Shows the number of relevant articles published in different countries and regions and the cooperation relationship. The size of the circle represents the number of articles published, the number of lines represents the number of cooperation, and the thickness of the lines represents the closeness of cooperation. **(B)** Shows the number of published articles and the cooperative relationship between different research institutions, and **(C)** shows the number of published articles and the cooperative relationship between different researchers.

Totally, of the 68 countries/regions of the 1,088 documents, 34 countries/regions met the requirement of ≥ 5 publications. Of the 34 countries/regions, 32 countries/regions had connections with others and constructed the network as Figure 3A. The 32 countries/regions were divided into 5 clusters in different colors, the size of circles reflected the publications of each region, and the size of lines reflected the link strength between the two regions. The top 10 high-yield regions are listed in Table 3. USA accounted for the most publications, followed by China, which might enter the field of PH non-invasive assessment relatively late but rapidly developed. Moreover, according to the results of co-authorship, the red cluster with the center of Italy (publications of 119), France (publications of 113), and England (publications of 72) was the largest (items of 11). The United States and France had the most cooperation partners of 26.

**TABLE 3:** Top 10 high-yield countries and regions/top 10 high-production organizations.

Rank	Countries/Regions	Documents	Citations	Avg. Pub. Year	Organization	Documents	Citations	Avg. Pub. year
1	United States	169	10,561	2014.567	University of Barcelona	31	2,298	2012.83
2	China	144	2,284	2017.31	Hospital Clinic de Barcelona	29	2,922	2013.69
3	Italy	119	6,815	2013.303	University of Bologna	28	1,308	2014.25
4	France	113	6,807	2013.441	Mayo Clinic	27	1,333	2014.37
5	Japan	93	2,613	2013.835	Yonsei University	27	883	2013.577
6	Spain	90	8689	2013.966	Medical University of Vienna	26	801	2017.24
7	Germany	82	2747	2014.45	University of Bern	22	818	2018
8	England	72	2128	2015.268	University of Milan	19	909	2012.79
9	South Korea	60	2247	2015.068	Hopital Universitaire Beaujon	18	742	2015.529
10	Switzerland	40	1150	2017.8	University College London	18	410	2016.5

Totally, of the 1,400 organizations, 100 organizations met the restriction condition of publishing over 5 pieces of literature, of which 91 organizations had connections and consisted of the co-authorship network (Figure 3B). The 91 organizations were grouped into 10 clusters, the size of circles reflected the publications of each organization, and the size of lines reflected the link strength between the two organizations. The red cluster, which was made up of 21 organizations, was the largest one, and “Mayo Clinic” is in the center. “University of Bologna” (in yellow Cluster 4) and “University of Bern” (in blue Cluster 3) have the most cooperative organizations (*n* = 26). The top 10 high-production organizations are shown in Table 3.

### Co-authorship of authors

In total, there were 5,779 authors of 1,088 pieces of literature; 130 authors met the threshold of over 5 publications, of which, 100 authors have a connection and composed the co-authorship network as Figure 3C. The 100 authors were grouped into 10 clusters, the size of circles reflected the publications of each author, and the size of lines reflects the cooperation strength between the two authors. The red Cluster 1 with the center of “berzigotti, annalisa” included 17 authors and was the largest cluster; “berzigotti, annalisa” was also the highest-yield author with 40 publications, had the most cooperation authors (*n* = 42), and had a connection with other 7 clusters (except for yellow Cluster 4 and pink Cluster 10). The top 10 high-yield authors are listed in Table 4, and the highest-yield author of each cluster is also listed in Table 5.

**TABLE 4:** Top 10 high-yield authors.

Rank	Author	Documents	Citations	Avg. citations	Avg. Pub. year
1	Berzigotti, Annalisa	40	2,172	54.3	2015.5
2	Castera, Laurent	23	3,690	160.4348	2013.739
3	Reiberger, Thomas	21	596	28.381	2018.1
4	Pinzani, Massimo	19	1,963	103.3158	2014.5
5	Bosch, Jaime	17	1,509	88.7647	2013.118
6	Abraldes, Juan G.	16	1,140	71.25	2013.313
7	Festi, Davide	16	727	45.4375	2017.188
8	Mandorfer, Mattias	15	365	24.3333	2019
9	Ehman, Richard L.	14	1,025	73.2143	2014.357
10	Procopet, Bogdan	14	902	64.4286	2015.429

**TABLE 5:** The highest-yield author of each cluster.

Cluster	Author	Documents	Citations	Avg. citations	Avg. Pub. year
1	Berzigotti, Annalisa	40	2,172	54.3	2015.5
2	Reiberger, Thomas	21	596	28.381	2018.1
3	Pinzani, Massimo	19	1,963	103.3158	2014.5
4	Ehman, Richard L.	14	1,025	73.2143	2014.357
5	Castera, Laurent	23	3,690	160.4348	2013.739
6	Sporea, Ioan	11	634	57.6364	2015.182
7	Han, Joon Koo	9	383	42.5556	2016.111
8	Qi, Xiaolong	7	87	12.4286	2019.333
9	Procopet, Bogdan	14	902	64.4286	2015.429
10	Kleiner, David E.	7	171	24.4286	2016.571

### Co-citation of documents

In total, there were 20,558 references of 1,088 retrieved pieces of literature, from 2,908 journals, having 13,108 first authors. Of the 20,558 references, there were 309 references cited over 20 times by the 1,088 retrieved literature. The top 10 high-cited references are listed in Table 6. The 10 documents were all articles (no review) and are all cited over 100 times. “Liver stiffness measurement predicts severe portal hypertension in patients with HCV-related cirrhosis” published in 2007 was the highest cited (*n* = 254) and was often co-cited with “Transient elastography accurately predicts the presence of significant portal hypertension in patients with chronic liver disease” (rank 8th) published in 2008, “Liver stiffness measurement selects patients with cirrhosis at risk of bearing large esophageal varices” (rank 7th) published in 2006, and “Transient elastography for diagnosis of advanced fibrosis and portal hypertension in patients with hepatitis C recurrence after liver transplantation” (rank 14th) published in 2006. Moreover, “Expanding consensus in portal hypertension: Report of the Baveno VI Consensus Workshop: Stratifying risk and individualizing care for portal hypertension” (rank 2nd) was more recent.

**TABLE 6:** The top 10 high-cited references.

Rank	First author	Title	Journal	Year	Citations
1	Vizzutti, Francesco	Liver stiffness measurement predicts severe portal hypertension in patients with HCV-related cirrhosis	Hepatology	2007	254
2	de Franchis, Roberto	Expanding consensus in portal hypertension: Report of the Baveno VI Consensus Workshop: Stratifying risk and individualizing care for portal hypertension	Journal of Hepatology	2015	209
3	Wai, Chun Tao	A simple non-invasive index can predict both significant fibrosis and cirrhosis in patients with chronic hepatitis C	Hepatology	2003	190
4	Sandrin, Laurent	Transient elastography: a new non-invasive method for assessment of hepatic fibrosis	Ultrasound in Medicine and Biology	2003	179
5	Colecchia, Antonio	Measurement of spleen stiffness to evaluate portal hypertension and the presence of esophageal varices in patients with HCV-related cirrhosis	Gastroenterology	2012	167
6	Castera, Laurent	Prospective comparison of transient elastography, Fibrotest, APRI, and liver biopsy for the assessment of fibrosis in chronic hepatitis C	Gastroenterology	2005	164
7	Kazemi, Farhad	Liver stiffness measurement selects patients with cirrhosis at risk of bearing large esophageal varices	Journal of Hepatology	2006	157
8	Bureau, Christophe	Transient elastography accurately predicts presence of significant portal hypertension in patients with chronic liver disease	Alimentary Pharmacology and Therapeutics	2008	151
9	Giannini, Edoardo Giovanni	Platelet count/spleen diameter ratio: proposal and validation of a non-invasive parameter to predict the presence of esophageal varices in patients with liver cirrhosis	Gut	2003	144
10	Ziol, Marianne	Non-invasive assessment of liver fibrosis by measurement of stiffness in patients with chronic hepatitis C	Hepatology	2005	144

### Co-occurrence of all keywords

**FIGURE 4:**
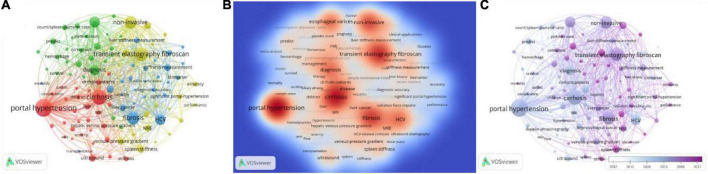
The co-occurrence analysis of keywords occurred over 20 times. **(A)** Shows the number of times the keywords appear and how they relate to one another. The size of the circles indicates how often they appear, the lines between the circles indicate how connected they are, and the thickness of the lines indicates how close the relationship is. **(B)** Shows the current research hotspots in keywords, and the red circles represent the research hotspots. **(C)** The color of the timeline in the lower right corner represents the year in which the keywords appeared.

There was a total of 2,806 keywords retrieved from 1,088 publications. Among them, 102 keywords occurred over 20 times and formed the network as Figure 4A. The keywords density visualization map is shown in Figure 4B. The top 25 high-occurrence keywords are listed in Table 7. The order of clusters depended on the number of keywords in each cluster. The 102 keywords were divided into 4 clusters; the most frequent keywords in Clusters 1, 2, 3, and 4 were “portal hypertension,” “esophageal varices,” “fibrosis,” and “transient elastography fibroscan,” respectively. According to the result of the analysis, elastography based on ultrasound or magnetic resonance accounted for the most important role in predicting portal hypertension.

**TABLE 7:** Top 25 high-occurrence keywords.

Rank	Label	Cluster	Occurrences	Avg. Pub. year
1	Portal hypertension	1	744	2014.951
2	Cirrhosis	1	557	2014.846
3	Transient elastography fibroscan	4	461	2015.653
4	Fibrosis	3	400	2015.463
5	Non-invasive	4	389	2015.479
6	Esophageal varices	2	298	2015.449
7	HCV	3	275	2014.434
8	Diagnosis	2	246	2014.284
9	Liver stiffness	4	138	2016.587
10	Disease	3	132	2014.385
11	Spleen stiffness	4	131	2017.177
12	Ultrasound	1	131	2013.74
13	Elastography	4	130	2017.242
14	Risk	2	119	2016.931
15	Stiffness measurement	3	119	2015.496
16	Liver cancer	3	113	2015.67
17	MRE	4	111	2016.418
18	Venous-pressure gradient	4	110	2015.43
19	Management	2	104	2015.748
20	Predict	2	97	2014.691
21	Shear wave elastography	4	95	2018.297
22	Liver	1	91	2015.067
23	Liver stiffness measurement	3	87	2015.279
24	Biopsy	3	83	2013.317
25	Gastroesophageal varices	2	74	2016.493

### Co-occurrence overlayed with time

Figure 4C shows the development trend of keywords in recent years. The timeline in the lower right corner of the picture showed the years represented by different colors. As technology advances, the research hotspots of researchers and research institutions also change over time. It could be clearly seen from the figure that the hotspots of the current research were transient elastography fibroscan, liver and stiffness hardness, and stiffness measurement, while Doppler ultrasonography was a relatively old topic. This indicated that elastography still attracted the major attention of researchers in this field today.

## Discussion

### Blue cluster 3: The evaluation of portal hypertension based on fibrosis

**FIGURE 5:**
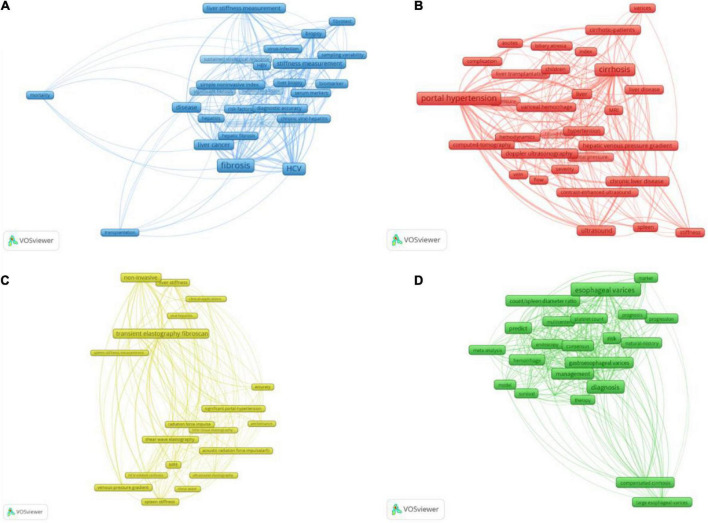
The four clusters of the 102 keywords occurred over 20 times. The size of frames represented the occurrence times of the keywords, and the line thickness indicated the link strength between the keywords at either end. Panel **(A)** is Cluster 3 about the evaluation of portal hypertension based on fibrosis. This cluster included 26 keywords, and “fibrosis” was the highest frequency. The keywords relating to the method of PH evaluation were “liver stiffness measurement,” “fibrotest,” “stiffness measurement,” “biomarker,” “serum markers,” and “platelet ratio index.” Panel **(B)** was Cluster 1 about the evaluation of portal hypertension based on hemodynamic factors. There were 30 keywords, and “portal hypertension” was the highest frequency. The referred indices included “blood-flow,” “hemodynamics” and “ascites,” and referred methods were “ultrasound,” “Doppler ultrasonography,” “contrast-enhanced ultrasound,” “computed-tomography” (CT), and “MRI.” Panel **(C)** was Cluster 4 about the evaluation of portal hypertension through elastography. There were 20 keywords and “transient elastography fibroscan” was the highest frequency. Elastography could be divided into “ultrasound elastography” and “MRE,” and “transient elastography fibroscan” was the most commonly used method of “ultrasound elastography.” Panel **(D)** Was Cluster 2 about the evaluation of portal hypertension-induced variceal bleeding. There were 26 keywords and “esophageal varices” was the highest frequency. This cluster is also referred to “platelet count” and “count/spleen diameter.”

Blue Cluster 3 consisted of 26 keywords shown in Figure 5A, mainly about “the evaluation of portal hypertension based on fibrosis.” In this cluster, the highest-occurrence word was “fibrosis,” and the studied populations included the patients with “HCV” (occurrence of 275), “HBV” (occurrence of 57), and “non-alcoholic fatty liver disease” (occurrence of 23). The keywords “liver stiffness measurement” and “stiffness measurement” worked as biomechanical indexes of fibrosis to evaluate portal hypertension and would be addressed in more detail in Cluster 4, while the keywords “biomarker,” “serum markers,” “platelet ratio index,” and “fibrotest” reflected the application of biochemical markers to assess liver fibrosis and portal hypertension, which is described in detail in this part. Around this theme, we also further retrieved and supplemented the information of other detecting methods based on fibrosis.

Portal hypertension-related fibrosis included liver fibrosis and spleen fibrosis, the former was the leading component of PH, and the latter reflected the extrahepatic secondary alterations caused by PH. This part mainly depicted the PH evaluation based on liver fibrosis. Liver fibrosis was characterized by the chronic inflammatory insult of liver parenchyma and excessive collagen deposition and was also accompanied by the occurrence of endothelial dysfunction (25). Cirrhotic PH was determined by intrahepatic collagen deposition, increased intrahepatic vascular tone, and extrahepatic hyperdynamic circulation. Excessive collagen accumulation was the major component of portal hypertension (PH), accounting for 70–80% (26). Therefore, evaluating PH based on the level of fibrosis was rational and reliable.

#### Blood test

##### The indicators of liver function

The injury of liver parenchyma was the feature of liver fibrosis, related liver function indices including single platelet count (PLT), aspartate aminotransferase to alanine aminotransferase (AAR) ratio, aspartate aminotransferase to platelet count ratio index (APRI), fibrosis index based on 4 factors (FIB-4 consists of PLT, AST, ALT, and age), glutamyl transpeptidase to platelet (GPR), King’s score (consists of Age, AST, INR, and PLT), Lok score (consists of PLT, AST, ALT, and INR), and the CSPH risk score (consists of ALB, INR, and ALT) have been validated to evaluate PH. Using HVPG ≥ 10 mmHg as the golden standard, the area under the receiver operating characteristic curve (AUROC) of the above indices to diagnose CSPH was 0.6–0.8 and hardly exceeds 0.8 (27–29).

##### The indicators of collagen deposition

In addition to the above conventional biomarkers, the more proprietary markers like increased osteopontin (OPN) accompanying liver fibrosis have also been discussed (30). Plasma OPN>80 ng/ml could detect CSPH with a sensitivity of 75% and specificity of 63%, and plasma OPN>90 ng/ml could detect HVPG ≥ 12 mmHg with a sensitivity of 71% and specificity of 62%. Similarly, apelin secreted by activated hepatic stellate cells showed the potency to predict CSPH with AUROC of 0.962 (31).

A recent study (32) of NASH cirrhosis has shown that the enhanced liver fibrosis (ELF) score correlates with HVPG at values<20 mmHg. The ELF score includes three indices: tissue inhibitor of matrix metalloproteinases (TIMP1), the aminoterminal peptide of procollagen type III (PIIINP), and hyaluronic acid (HA), which mainly reflects the stroma deposition during liver fibrosis. ELF identified patients with a high probability of CSPH with AUROC of 0.833; however, the performance of ELF to detect HVPG > 20 mmHg is unsatisfactory with AUROC of 0.677. Moreover, ELF could also detect CSPH with AUROC of 0.884 in the patients with recovered HCV after 12 months of sustained virological response (33).

##### The indicators of endothelial dysfunction

An important determinant of increased intrahepatic vascular resistance was endothelial dysfunction (34). Asymmetric dimethylarginine (ADMA) is an endogenous nitric oxide synthase inhibitor and a recognized mediator and marker of endothelial dysfunction. A statistically significant positive correlation was found between ADMA and portal hypertension (*R* = 0.77, *p* < 0.0001) in 2007, but missed further study (35). One study (36) published in 2012 supported that von Willebrand factor antigen (vWF-AG) released by activated endothelial cells (ECs) had the potential to be a novel biomarker to monitor PH. This study in 286 patients showed that vWF-Ag was correlated with HVPG (*R* = 0.69, *p* ≤ 0.0001) and was independent of the Child-Pugh score prediction. When the taken cut-off value of vWF-AG was ≥ 241%, the positive predictive value of detecting CSPH was 87%, and the negative predictive value was 80% (AUROC, 0.85). Another study (37) combining vWF-AG and PLT (VITRO) reported better detection of CSPH with AUROC of 0.86. Ding et al. (38) conducted a meta-analysis on the relationship between the vWF and portal hypertension. The results showed that the serum vWF in the cirrhosis group with portal hypertension was significantly higher than that in the non-portal hypertension group, with a comprehensive sensitivity of 0.823 (95% CI:0.788, 0.855) and a binding specificity of 0.782 (95% CI:0.708, 0.845). The diagnostic odds ratio was 18.347 (95% CI: 11.725, 28.708), and the AUROC was 0.8896. Others, the meta-analysis by Zou et al. (39) also proved that vWF was moderately correlated with HVPG and performed well in the diagnosis of CSPH and SPH in patients with cirrhosis. But EASL Clinical Practice Guidelines considered that the validation studies of vWF were still insufficient (40).

##### The indicators of inflammation

Inflammation was an initiating and accompanying factor of liver fibrosis, and the study (41) in 90 cirrhotic patients proved the significant correlation between PH and inflammatory biomarkers, including IL-1b, IL-1Ra, Fas-R, and VCAM-1, and construct a paradigm based on TNFb, HSP-70, alcohol use, and Child-Pugh Class B, which could screen HVPG < 12 mmHg with the accuracy of 86%, however, performed poorly in diagnosing HVPG ≥ 12 mmHg with AUROC of 0.767.

Under the stimulation of long-term inflammation, liver macrophages would be activated to varying degrees (42). CD163, a scavenger receptor expressed only in monocytes and macrophages, was upregulated and entered the blood circulation as soluble CD163 (sCD163) under macrophage proliferation and activation conditions (43). During cirrhosis, Kupffer cells were also activated in patients with cirrhosis and portal hypertension. They showed more than three times the concentration of sCD163 in patients compared with the control group (median, 5.22 mg/L vs. 1.45 mg/L, *p* ≤ 0.001) (44). On this basis, Grønbaek et al. (45) measured sCD163 concentrations, HVPG values, cardiac output (CO), cardiac index, and systemic vascular resistance (SVR) in 81 cirrhotic patients (26 Child A patients, 29 B patients, and 26 C patients) and 22 healthy subjects. When the concentration of sCD163 reached 5 mg/L, HVPG increased sharply, with an asymptote of 22 mmHg. When the concentration of sCD163 increased further, HVPG did not increase. The results suggested that sCD163 was an independent predictor of HVPG. Sandahl et al. (46) predicted portal hypertension by combining sCD163 values with the ELF score. The AUROC of HVPG > 10 mmHg predicted by a single indicator was about 0.80, and the combined score optimized by logistic regression analysis improved the AUROC to 0.91 in the test cohort and 0.90 in the validation cohort. These results indicated that the combination of macrophage activation marker sCD163 and collagen accumulation markers could predict significant portal hypertension with higher accuracy.

The changes and shifts of intestinal flora caused by cirrhosis and portal hypertension had always been a research hotspot, but this phenomenon had not been thoroughly analyzed in PH. A recent study from 2022 (47) has shown that the circulating plasma microbiota profile of cirrhotic patients was significantly different from that of the control group, characterized by rich *Comamonas*, *Cnuella, Dialister*, *Escherichia/Shigella*, and *Prevotella*, but poor *Bradyrhizobium*, *Curvibacter*, *Diaphorobacter*, *Pseudarcicella*, and *Pseudomonas*. There was no significant difference in peripheral and hepatic venous blood in patients with cirrhosis. Concentrations of *Bacteroides*, *Escherichia/Shigella*, and *Prevotella* were found in patients with severe portal hypertension (SPH). Another study (48) had shown that short-chain fatty acids (SCFAs), a derivative of intestinal microorganisms involved in maintaining the integrity of the intestinal barrier and the host immune response, had a negative correlation with HVPG values. The blood level of SCFA in patients with cirrhosis showed an overall trend of decline. Although the composition of circulating microorganisms could not predict the severity of PH at present, it might be studied as a future direction of research.

In general, routine blood tests were minimally invasive, convenient, and reproducible; however, the PH predictive accuracy of these biomarkers did not meet the clinical need, and they were easy to be affected by the various systemic disease. As suggested by EASL Clinical Practice Guidelines and Baveno VII (17, 40), serum markers solely were insufficient to detect PH but could work as a complement.

##### Computed-tomography: Liver surface nodularity

In addition to the biochemical markers, CT and magnetic resonance imaging (MRI) could also assess the level of fibrosis through related morphological features, and then evaluate PH. Liver surface nodularity (LSN) has been proven to differentiate cirrhotic from non-cirrhotic livers with AUROC = 0.929 (49). Based on the high correlation between liver fibrosis and PH, Sartoris et al. (50) showed the probability to evaluate PH by quantifying LSN in CT images. According to the CT images on the portal venous phase, the LSN score was calculated by measuring the roughness of the real liver margin using a smooth polynomial line that delineated the normally assumed surface margin of the left liver. The LSN scores of patients with CSPH were higher than that of non-CSPH patients (3.2 ± 0.6 vs. 2.4 ± 0.3; *p* < 0.001) and correlated with HVPG (*r* = 0.75, *p* < 0.001). When the LSN scores cutoff value of 2.8 was used to detect CSPH, the positive predictive value was 88%. The AUROC of LSN scores was 0.88 ± 0.03, which was higher than most blood tests. LSN scores could also be obtained from MRI, and the result was similar (51). But the sample size of the two studies was small.

#### Magnetic resonance imaging-mapping

In 2014, MR T1 mapping of the liver was firstly reported to provide high diagnostic accuracy in the non-invasive assessment of liver fibrosis (52). Extracellular volume fraction (ECV) was a measure of the extracellular space and represented the percentage of the volume of non-cellular tissue. T1 values could be measured before and after the extracellular contrast agent was given to calculate the extra computed extracellular volume (ECV) (53). In 2018, Luetkens et al. (54) designed animal studies to validate the non-invasive assessment of liver fibrosis and portal hypertension by evaluating MRI T1 and T2 positioning techniques. Results showed that T1 and T2 values were significantly higher in the bile duct ligation (BDL) model and carbon tetrachloride (CCl4) intoxication than in the control group (*p* < 0.001). ECV values showed the same trend. The results showed a high correlation between ECV and portal hypertension (BDL: *r* = 0.54, *p* = 0.003; CCl4: *r* = 0.39, *p* = 0.043). Subsequently, clinical data by Mesropyan et al. (55) showed that splenic ECV correlated with portal pressure (*r* = 0.72; *p*<0.001) and directly with HVPG (*r* = 0.50; *p* = 0.003), and had a good diagnostic effect for CSPH (AUC = 1.000). In the validation cohort, the diagnostic performance of mapping parameters was comparable. Splenic ECV has a good predictive value for portal hypertension in patients with advanced liver disease.

#### Artificial intelligence/computational model

Artificial intelligence and machine learning, which seem to have nothing to do with medicine, have been gradually changing clinical diagnosis and treatment by fully utilizing the information from routine imaging, serological, and other examinations. Bosch et al. (56) conducted a study to measure portal pressure in patients with NASH cirrhosis using machine learning (ML). The results showed that the identification ability of the model was higher than that of liver collagen (AUROCs in the test set:0.76 vs. 0.65) and similar to the ELF score (AUROC, 0.78) when used alone. The ML model could identify fibrous matrix features associated with increased portal pressure. Furthermore, the complete model combining the ML model with the conventional clinical parameters (ELF, platelet, AST, bilirubin, APRI, and FIB-4) improved the performance of the ML HVPG score in detecting CSPH (AUROC of the test set ranging from 0.81 to 0.85). However, in the case of higher portal pressure, the correlation between the algorithm and HVPG was weak. The ML HVPG score did not predict clinical events at the baseline, possibly due to the ML algorithm’s inability to distinguish between portal pressure levels above the CSPH threshold.

In summary, the ML algorithm could identify fibrous matrix features and correlate with HVPG measurements. In addition to morphology-based features, texture features and deep convolutional neural network-based imaging analysis also showed a potential performance in assessing PH (28, 57). However, due to the end-to-end nature of the model, specific features that helped improve diagnostic performance could not be elucidated.

### Red cluster 1: The evaluation of portal hypertension based on hemodynamic factors

Red Cluster 1 includes 30 keywords shown in Figure 5B mainly about “the evaluation of portal hypertension based on hemodynamic factors,” the mentioned methods included “ultrasound,” “Doppler ultrasonography” (DUS), “MRI,” “computed-tomography” (CT), and “contrast-enhanced ultrasound” (CEUS), which could monitor the blood flow of portal system directly or through the phase change of contrast. CEUS was relatively recent, and its average publication year was 2016.

The PH assessment methods based on fibrosis and hemodynamics were not completely separate, as hemodynamic indices like hepatic perfusion could also be used to evaluate the level of liver fibrosis. PH-related hemodynamic factors include intrahepatic and extrahepatic blood flow, the former mainly included portal vein, hepatic vein, and hepatic artery; while the latter included splenic vein, renal artery, and collateral circulations. US, CT, and MRI could provide static indices as vessel diameter and dynamic indices as flow velocity to reflect portal pressure. Moreover, ascites, spleen sclerosis, and splenomegaly could also be regarded as extrahepatic hemodynamic factors.

#### Blood test

Although imaging was the most common method of estimating blood flow, blood tests like the filtration test of indocyanine green (ICG) and cholate could also reflect the status of hepatic blood flow, which worked as the contrast in imaging. The ICG retention test was first used to evaluate the residual liver function before hepatectomy and was determined by the liver secretory function and liver blood flow (58). In 2014, one study (59) suggested the indocyanine green 15-min retention (ICG-r15) test as a non-invasive method of monitoring PH. To exclude the effect of impaired liver function, all 96 participants were in the child grade A (74 with clinically significant CSPH and 59 with SPH); ICG-r15 values ≥ 16.7% and ≥ 18.4% could detect CSPH and SPH, respectively, with the high specificity of 90.9 and 91.9% but with relatively low sensitivity of 60.8 and 62.7% (AUROC, 0.808 and 0.821). The same authors also conducted prospective studies to demonstrate that ICG-r15 was significantly related to PH and could predict the occurrence of hepatic decompensation (60). Another research (61) further studied the application of ICG-r15 in patients with different child grades and found that the child grade did impact the diagnostic power of ICG-r15. In Child A, ICG-r15 was more correlated with CSPH than in Children B and C (AUROC, 0.832 vs. 0.7448 vs. 0.7392). Moreover, the previous research had found that the PH of alcohol-related cirrhosis was higher than that of virus-related cirrhosis with similar residual liver function. A recent study (62) in the cirrhotic patients of Children A and B has shown that the ICG-r15 level was correlated with portal pressure in both non-viral cirrhosis and viral cirrhosis, which could detect HVPG ≥ 12 mmHg with the specificity of 79.0% and sensitivity of 72.3% (AUROC, 0.780).

Cholate, similar to ICG, was high hepatic extraction, and its extraction was determined by hepatic blood flow instead of hepatic metabolism (63). The more recent HepQuant SHUNT trial has used double clearance of cholates after oral and intravenous administration to quantify hepatic perfusion without the effects of total plasma volume. A study (29) of early clinical compensatory chronic liver disease in 42 patients found that patients with portal hypertension had lower portal hepatic filtration rates and higher portal-systemic spillover of oral d4-cholate percentage (SHUNT%), which presented the increasing portal-systemic flow and the decreasing intrahepatic flow. When evaluated CSPH, using HVPG > 10 mmHg or direct portal pressure (dPP) > 22 mmHg as gold standards, the sensitivity and specificity of SHUNT% > 0.35 was 76.9 and 89.6%, respectively. Another key finding was that SHUNT% was able to detect portal hypertension early in the course of disease in patients without cirrhosis, as SHUNT% > 0.295 could detect HVPG > 5/dPP > 17 mmHg, with the sensitivity of 85.7% and specificity of 80.9%. This indicated that HepQuant SHUNT trial results were significantly positive at portal pressure ≥ 5 mmHg. The non-invasive Hepquant SHUNT assay, either alone or in combination with other non-invasive tests, could be an alternative to existing invasive methods for assessing portal vein pressure and hypertension.

Although the principle was similar to ICG, there was no comparison between the HepQuant SHUNT test and ICG-R15 in predicting portal pressure. Additionally, the relatively complicated and costed blood tests, such as ICG-r15 and HepQuant SHUNT tests, required larger confirmatory studies to check the cut-offs and the feasibility in different clinical scenarios.

#### Doppler ultrasonography and contrast-enhanced ultrasound

The introduction of Doppler ultrasound (DUS) was an important breakthrough in evaluating organ hemodynamics (64). DUS was used early as a non-invasive tool to predict portal hypertension, which could provide real-time hemodynamic information, like (i) the velocity of the portal vein (↓), splenic vein (↑), and azygos vein (↑), (ii) resistive or pulsatility indices of the hepatic artery (↑), splenic artery (↑), and renal artery (↑), and (iii) hepatic vein waveform/hepatic vein waveform damping index (65). As early as 1997, researchers found that the hepatic vascular index (portal vein velocity/hepatic arterial pulsatility indices) was a highly sensitive and specific DUS parameter for diagnosing liver cirrhosis and portal hypertension, and liver cirrhosis and portal hypertension could be identified when the hepatic vascular index was less than 12 cm/s (66). Berzigotti et al. (67) examined the correlation between renal vascular impedance measured by pulsed DUS and portal pressure measured by HVPG in patients with cirrhosis and found that, in cirrhotic patients with normal renal function, high impedance of renal vasoconstriction could be considered an indication of HVPG ≥ 16 mmHg, although CSPH might have normal renal atrial resistance. Routine examination of renal vascular impedance in patients with cirrhosis might help non-invasively identify a group of patients with portal hypertension at high risk for complications. Berzigotti et al. also found that the emergency abdominal collaterals examined by DUS could detect HVPG ≥ 16 mmHg with a sensitivity of 57% and a specificity of 87%. They also evaluated the prognostic value of a single HVPG measurement and DUS in patients with cirrhosis and CSPH. Although HVPG had a strong and independent predictive value for mortality and first decompensation in patients with cirrhosis and CSPH, DUS to examine abdominal vessels still had a high positive predictive value (68). Another hepatic vein waveform, reflecting the change of central venous pressure during the cardiac cycle, should be a normal triphasic waveform. The portal-hepatic vein shunts accompanying portal hypertension could cause the flattening of the hepatic vein waveform. A study (69) found a correlation between the abnormal hepatic venous waveform and HVPG in patients with liver cirrhosis. There were 44 cases (56%) having biphasic waveforms, 28 (36%) having monophasic waveforms, and 6 (8%) having triphasic waveforms. The monophasic waveform was associated with HVPG > 15 mmHg, with a sensitivity of 74% and a specificity of 95%. Furthermore, the damping index (DI), equaling minimum velocity divided by the maximum velocity of the hepatic vein waveform, was a quantitative index of the hepatic vein waveform. DI > 0.6 could detect HVPG > 12 mmHg, with a sensitivity of 75.9% and a specificity of 81.8% (70).

Contrast-enhanced ultrasound (CEUS) with microbubbles improved the imaging quality of the US with a higher signal-to-noise ratio. When the ultrasound pulse reached the microbubbles, the microbubbles resonated and scattered the fundamental wave, subharmonics, second harmonics, and superharmonics, and the harmonics created contrast imaging with tissue echoes. Among these harmonics, the second harmonic was the strongest signal (71). The hemodynamic indices of CEUS could be divided into two types: (i) the transit time of microbubbles and (ii) the size change of the microbubbles. Berzigotti et al. (72) validated the inverse correlation of regional hepatic perfusion (RHP, as microbubbles velocity × microbubble concentration) and effective perfusion of the liver, which proved that RHP increased primarily through intrahepatic shunts caused by PH. Studies using CEUS to assess portal pressure have been carried out on this basis. Kim et al. (73) explored the correlation of HVPG and hepatic vein arrival time (HVAT, transit time from venous injection to arriving hepatic vein) in 71 consecutive patients with compensated cirrhosis (derivation set) and validated it in 35 patients of another medical center (validation set). For CSPH, the sensitivity and the specificity of HVAT < 14 s were, respectively 92.7 and 86.7%. In the validation set, HVAT was still highly correlated with HVPG; the AUROC of detecting CSPH was 0.953. The study by Jeong et al. (74) showed that intrahepatic transit time was significantly reduced in the SPH group (HVPG ≥ 12 mmHg) compared to the non-SPH group. ITT ≤ 6 s could indicate HVPG ≥ 12 mmHg with a sensitivity of 92% and a specificity of 89%. Moreover, the CEUS parameter about splenic circulation was also evaluated to assess PH based on the increasing resistance of splenic vessels following PH. A study (75) in 62 patients with liver cirrhosis and 29 patients in the control group found that peak enhancement time (PET, transit time from onsetting in splenic artery to reaching maximum intensity in splenic vein) could also identify HVPG ≥ 10 mmHg and ≥ 12 mmHg with AUROC of both 0.76.

The received intensity of the ultrasound signal was determined by transmitting pulses and microbubble scatter areas. Due to the compressibility of a microbubble, ambient pressure could change its size and scatter area. Hence, the change of harmonic amplitude could reflect ambient pressure, and the subharmonic was the most sensitive harmonic (76). Based on the above, subharmonic-aided pressure estimation (SHAPE, the mean subharmonic signal in the hepatic vein minus that in the portal vein) was used to evaluate PH. In 2013, Eisenbrey et al. (77) conducted a pilot study of SHAPE in 33 patients with chronic hepatitis. They validated the good correlation of SHAPE and HVPG (*r* = 0.82), and the correlation was greater (*r* = 0.97) when HVPG ≥ 12 mmHg (*n* = 6). In 2021, a larger-scale clinical study in 125 patients with chronic hepatitis of various etiologies was carried out by Gupta et al. (78). Compared to the pilot study, the correlation decreased (*r* = 0.68), and the correlation was lower (*r* = 0.41) when the HVPG ≥ 12 mmHg (*n* = 14), which was contradictory and possibly because of the low sample size or the changed transmit pulses. Nonetheless, SHAPE ≥ −0.11 dB could discriminate HVPG ≥ 10 mmHg with sensitivity of 91% and specificity of 82% and discriminate HVPG ≥ 12 mmHg with sensitivity of 90% and specificity of 80%.

Generally, although DUS was easy to use and could acquire data in real-time with non-invasiveness, it was not easy to measure due to the influence of fat, ascites, and the depth of targeted vessels. Low accuracy, large variability, and a certain failure rate of DUS limited the prediction of PH. Hence, the study of single parameters of DUS has been less and less in recent years, while CEUS emerged as the updated way, which could provide clearer and more accurate signals. SHAPE was a special and novel application of CEUS and was suggested to do further validation by the Baveno VII. But CEUS was limited by the availability of contrast agents and is still not available in some countries. The dynamics and metabolism of microbubbles *in vivo* had not been fully studied, and the interpretation of the contrasting results also required further study (79). Besides, CEUS still has the inherent disadvantages of the US and needs more validation research.

#### Computed-tomography

Recently, an increasing number of studies have explored, applying CT or MRI to assess PH. CT or MRI could provide both the hemodynamic and morphological information, which was more stable and consistent than that provided by the US. Moreover, compared with the point information measured by the US, CT, and MRI could provide the hemodynamic information of the total portal system.

In 2014, Iranmanesh et al. conducted a study (80) on 65 patients with hepatocellular carcinoma (HCC) and showed that the liver/spleen volume ratio and peri-hepatic ascites in contrast-enhanced multi-detector CT were good predictors for PH. The statistical models based on these two indices, HVPG score = 17.37–4.91 × ln (Live/Spleen volume ratio) + 3.8 (if with peri-hepatic ascites), were proved to predict HVPG > 10 mmHg with a sensitivity of 92% and specificity of 79% (AUROC, 0.911), when the cut-off was 11.606. Furthermore, in the validation set of 70 patients (the number of HCC patients was 24), the HVPG score could still assess PH with an AUROC of 0.820. Afterward, there were other studies about non-invasive methods of PH monitoring using this model as the control, and the AUROC of the HVPG score in these studies was < 0.8 (50, 81). Qi et al. (82) validated this model in 131 patients with HBV-related cirrhosis and concluded that the HVPG score was not enough to diagnose CSPH with AUROC of only 0.568. Iranmanesh explained that the difference in AUROC might be caused by the higher HVPG in Qi’s study (HVPG: 16.58 ± 5.84 mmHg vs. 11 ± 6 mmHg), and their model was more accurate when HVPG was around 10 mmHg (83). Overall, the HVPG score based on CT was unsatisfactory in the external validation and was not widely applicable to patients with *varying degrees* of PH.

#### Magnetic resonance imaging

In 2003, flow parameters measured by MRI had been studied to evaluate portal hypertension and were compared with DUS (84). Due to the inherent advantages of lower intra- and inter-observer variability, an increasing number of studies have focused on evaluating PH by using blood flow parameters measured by MRI instead of DUS in the recent past.

A study (85) in 30 patients with mean HVPG = 9.8 mmHg constructed a PH prediction model, combining hemodynamic information with liver fibrotic information measured by MRI. Based on the prior validation of the correlation between liver T1 relaxation time and liver fibrosis, this study found a good correlation between splenic artery velocity and HVPG and concluded that the best predictive model for HVPG was −28 + 0.04*(liver spin-echo echo-planar imaging T1) + 0.27* (splenic artery velocity). The calculated HVPG value based on this model kept a significant correlation with real HVPG (*R* = 0.85), and in the validation set with 10 patients still kept a high correlation. More recently, Hectors et al. (86) have collected information on 35 patients with cirrhosis measured by HVPG and examined using gadoxetate dynamic contrast-enhanced (DCE) MRI imaging of the liver and the spleen to investigate its potential for diagnosing portal hypertension. Liver ki (the intracellular uptake rate) showed the best diagnostic performance, with AUC, sensitivity, and specificity of 0.74, 71.4, and 78.6%, respectively. The combination of liver ki and spleen ve (Interstitial volume fraction) was the best method for diagnosing CSPH with an AUC of 0.87.

Besides, the velocity of azygos, hepatic arterial fraction of total liver blood flow, and portal vein hyperintensity on 20-min delayed T1 contrast-enhanced MRI observed by two-dimensional (2D) or three-dimensional (3D) MRI have also proved the correlation with HVPG (87–89), and azygos blood flow could detect HVPG ≥ 16 mmHg with AUROC of 0.96, while other indices lacked the further tests to validate their diagnostic performance.

Time-resolved 3D PC MRI with 3D flow velocity encoding referred to as 4D flow MRI allowed for the comprehensive *in vivo* measurement of 3D blood flow dynamics in the heart and the large vessels (90). Bane et al. (91) assessed 4D flow parameters for predicting the presence of cirrhosis/PH and the severity of the liver disease. The Spiral 4D flow provides a comprehensive assessment of the abdominal blood vessels in a single breath-holding activity and has considerable interobserver repeatability, but variability tests retest repeatability. Motosugi et al. (92) showed that azygos flowing greater than 0.1 L/min and portal vein flowing less than the sum of the splenic vein and superior mesenteric vein flow were useful markers of gastroesophageal variceal bleeding risk in patients with cirrhosis. Although 4D flow MRI has unique advantages, it still faces some problems in clinical applications. The first problem is that detection takes too long, such as 10–20 min for respiratory and cardiac Cartesian acquisition, which is the main reason preventing its clinical application (93). The second technical limitation of 4D flow is its need for a preset VENC parameter for measurements in all vessels captured within the acquisition volume. The VENC for 4D flow collection must be set according to the clinical problem being investigated, and multiple collections of different VENCs are required (94).

#### Artificial intelligence/computational model

In 2015, Amat-Roldan et al. (95) constructed a computational graph model of hepatic vascular network connectivity based on the imaging of CEUS in the right liver lobe. Due to the good capability of CEUS in microvessel imaging, this graph model could detect PH by reflecting the difference in the intrahepatic vascular network between normal and PH participants. In a healthy set (*n* = 4) with strong vascular network connectivity, the clustering coefficient was 0.4447; while, in the set with HVPG ≥ 10 mmHg (*n* = 11), the clustering coefficient was 0.0237. The correlation coefficient between the predicted HVPG of this model and measured HVPG was greater than 0.8. Xiao Long Qi and his team proposed an interesting idea (81), that was, to establish and validate a computed model of the HVPG based on CT vascular images, namely, virtual HVPG (v-HVPG), to achieve a non-invasive diagnosis of portal hypertension in liver cirrhosis. In brief, DUS and CT angiography were used to create a virtual HVPG based on a 3D reconstruction model and computational fluid dynamics. Using the original CT angiography images, a 3D model of the liver portal system was reconstructed from the 2D composite image and surrounding tissues, analyzed using the computational fluid dynamics solver ANSYS 13.0 (Ansys, Canonsburg, Pa), and meshed with inner tetrahedra. Portal venous velocity was measured by DUS, with portal venous velocity as the boundary condition. After that, finite element analysis and computational fluid dynamics analysis were used to calculate the pressure distribution of the 3D model. The average time to calculate the virtual HVPG was about 2.5 h, of which 1.5 h was spent on human processing and 1 h was spent on machine calculations. Results showed that, in the training cohort (*n* = 29), the AUROC of v-HVPG in predicting CSPH was 0.83. In the validation cohort (*n* = 73), diagnostic performance was prospectively confirmed with an AUROC of 0.89. The inter- and intra-observer agreement was 0.88 and 0.96, respectively, indicating good reproducibility of virtual HVPG measurements.

### Yellow cluster 4: The evaluation of portal hypertension through elastography

Yellow Cluster 4 consists of 20 keywords shown in Figure 5C, mainly about “the evaluation of portal hypertension through elastography.” Elastography was also based on fibrosis, so the locations of items in Cluster 4 and Cluster 3 were interleaved. Due to the great role in predicting portal hypertension and the large body of the related studies, elastography became a separate cluster. Elastography could be divided into “ultrasound elastography” (USE) and “MRE,” whose measure targets included “liver stiffness” and “spleen stiffness.” In USE, the “transient elastography fibroscan” was the most commonly used method, and “acoustic radiation force impulse (arfi),” including pSWE and 2D-SWE, was more recent than transient elastography.

#### Ultrasound elastography

Similarly, based on the relationship between liver fibrosis and PH, liver stiffness measurement (LSM) was the best recognized and the most widely used method to surrogate HVPG. The past study (96) had supposed to apply liver stiffness measured by transient elastography (TE) to evaluate PH more than a decade ago. After that, a series of studies focused on validating the feasibility and accuracy of LSM in different clinical settings, and a meta-analysis in 2017 showed that LSM with TE could detect CSPH with AUROC > 0.9 (97). Moreover, with the advancement of technology, point shear wave (pSWE) and two-dimensional real-time shear wave elastography (2D-SWE) emerged as a novel USE and were also validated to predict PH. Compared to TE, pSWE and 2D-SWE allowed flexibility in selecting the measured position and depth, and the region of interest (ROIs) of 2D-SWE was larger than that of TE and pSWE. However, in terms of the performance to discriminate liver fibrosis and PH, pSWE and 2D-SWE did not generally unfold the superiority (98). Due to the LSM of TE having the most studied data, the Baveno VI consensus firstly suggested that, in patients with virus-infected compensated advanced chronic liver disease (cACLD), LSM ≥ 20–25 kPa in TE could be regarded as CSPH. In contrast, patients with cACLD with other etiologies of PH required additional explanation (99). In addition, in the latest Baveno VII, LSM ≥ 25 kPa in TE of patients cACLD with virus hepatitis, alcohol-hepatitis, and NASH with BMI < 30 kg/m^2^ could be diagnosed as CSPH (17). Under the condition of the same LSM level, patients with NASH with BMI ≥ 30 kg/m^2^ showed a higher false-positive rate with a positive predictive value (PPV) of only 62.8%, which might derive from the technical accuracy influenced by the fat thickness and probe size (M or XL), or from the physical increased stiffness of fat accumulation, which lowers the correlation between liver stiffness and cirrhosis (100). Notably, this study also used HVPG as a golden standard to discuss the PH diagnostic ability of LSM. However, a recent study has supposed that HVPG may underestimate the real portal pressure of patients with NASH (22, 101), so the relationship between LSM and PPG in the patients with NASH should be further tested. As suggested by Baveno VII, the combination of LSM, platelet count, and BMI might be useful in the patients with NASH, but required further validation about proper technology, standards, and cut-offs. Otherwise, due to the relatively smaller body of studies and the variability of cut-offs among the various studies, LSM of pSWE and 2D-SWE was not recommended by the main guidelines of liver disease (17, 40). However, the Society of Radiologists in Ultrasound suggested that LSM>17 kPa (2.4 m/s) in pSWE and 2D-SWE could be used to screen CSPH (102).

Although LSM has gained widespread acknowledgment in assessing PH, the past study proved that the relationship between LSM and PH was blunted with increasing pressure when HVPG ≥ 10–12 mmHg, which might be from the stronger involvement of extrahepatic factors like collateral circulation (103). Moreover, after NSBB treatment, LSM had no significant decrease in the HVPG-down patients (104). Conversely, after etiological treatment, LSM might immediately decrease due to the regression of inflammation, while PH is still maintained (105). Hence, HVPG was still the only metric recommended by the clinical guidelines to evaluate the changed portal pressure after various treatments (17, 19).

Spleen stiffness measurement (SSM) holds the potential to address the above issues with the ability to reflect extrahepatic hemodynamic changes. In 2012, a study (106) in 100 patients with HCV with a median HVPG of 12 mmHg firstly validated the close relationship between SSM of TE and HVPG, and, compared to LSM, SSM owed a similar diagnostic capability to detect HVPG ≥ 10 mmHg, HVPG ≥ 12 mmHg with AUROC of 0.966 and 0.959, respectively. Moreover, SSM of TE had a similar effect to HVPG on predicting EV. One recent meta-analysis study has also shown the potential capacity of SSM in assessing CSPH, and the pool sensitivity and the specificity of SSM in 7 studies were 0.85 and 0.86, respectively (107). In the latest Baveno VII, SSM > 50 kPa of TE was regarded as a cut-off to identify CSPH. In assessing the changed portal pressure, SSM exhibited good values. In 2019, a prospective study (108) of 106 cirrhotic patients (mean HVPG = 19.6 ± 5.8 mmHg) in the derivation set and 63 patients (mean HVPG = 19.2 ± 4.9 mmHg) in the external validation set firstly validated that the difference of pre- and post-treatment spleen stiffness (ΔSS) measured by pSWE could be used to predict the hemodynamic change after NSBB therapy. Moreover, this study also constructed a model ΔSS (0.0490−2.8345 × ΔSS), which could screen the deceased HVPG ≥ 20% or the post-NSBB HVPG <12 mmHg with AUC of 0.801 and 0.848 in the derivation set and the external validation set, respectively. Another study (104) in 20 patients with high-risk EV also proved that the cutoff −10.5% of the changed SSM of TE could screen the deceased HVPG ≥ 10% with a sensitivity of 100% and specificity of 60% (AUROC.973), while the changed LSM do especially poorly (AUROC.587). Otherwise, there were also studies (109, 110) that showed that SSM was better than LSM in reflecting the changed portal pressure after TIPS and etiological treatments, but the data were relatively scarce.

In summary, USE was the most significant hot spot in predicting PH, which is comprehensively proper in terms of technical difficulty and risk, consumption of time and money, feasibility, and accuracy. The studies about USE in assessing PH could be mainly grouped into three directions:

1. The technical questions, including operation protocols (fast for 4 h, location, breath, probe size, measurement systems, etc.), the influence of subcutaneous fat thickness, spleen size, the alanine aminotransferase level, and other diseases.
2. The comparison of LSM and SSM
3. The combination of USE with other markers

For the first direction, there had been specialized recommendations (102, 111, 112) to standard operation for achieving the best reproducibility and consistency in the different studies. Furthermore, a new dedicated system for SSM was also studied (113), as the spleen was stiffer than the liver, and the upper limit of common TE may not cover the whole SSM. As supported by Baveno VII, the best cut-off of SSM by using a 100-Hz-specific TE-probe, pSWE, and 2D-SWE needed further validation. As for the second direction, in assessing untreated PH by LSM or SSM, neither was universally better than the other. Although SSM had superior performance in screening high-risk EVs than LSM, the successful measurement rate was lower than LSM, and the recognized cut-off SSM was still lacking (114, 115). There were also two studies (114, 116) discussing the sequential application of LSM and SSM, but the diagnostic accuracy has large gaps in these two studies. Additionally, in the case of reflecting the changed portal pressure after various treatments, SSM might be more potential than LSM. In the third direction, the combinations of (1) LSM of TE, spleen size of US, and PLT (LSPS) in untreated cirrhotic patients, (2) LSM of TE, sex, spleen size of US, and PLT (the PH risk score) in untreated cirrhotic patients, (3) LSM and VITRO in the patients after IFN-free therapy, (4) LSM and FIB-4 (PLT, AST, ALT, and age) in patients with chronic liver disease, all had been reported to diagnose CSPH with AUROC > 0.8, but also need large validation studies (27, 117, 118).

#### Magnetic resonance elastography

In addition to ultrasound elastography, MRE could also measure tissue stiffness in clinical MRI examinations by encoding shear wave propagation into magnetic resonance phase signals. MRE showed an excellent ability to detect liver fibrosis and cirrhosis with a low failure rate (119). The EASL Clinical Practice Guidelines mentioned MRE as the most accurate non-invasive approach to liver fibrosis staging (40). In a large animal model of cholestatic liver disease in 2013, Yin et al. (120) compared the association of MRE with direct portal vein pressure gradient (D-HVPG). The results showed that F1 fibrosis appeared in the liver of animals after 4 weeks, and the portal pressure reached 11. ± 5.1 mmHg; after 8 weeks, it became F3 fibrosis, and the portal pressure increased to 11.3 ± 3.2 mmHg. At the same time, the mean stiffness of the spleen increased from 1.72 ± 0.33 kPa to 3.54 ± 0.31 kPa after 4 weeks, while it stabilized at 3.38 ± 0.06 kPa at week 8, consistent with the pattern of changes in portal pressure. In a study on patients with HBV and HCV (121), researchers divided participants into:

• Liver biopsy with no evidence of portal hypertension (Group1, *n* = 155)
• Patients with portal hypertension who were tested for HVPG (Group 2, *n* = 85)
• Healthy subjects (Group 3, *n* = 60)

Among multiple viscoelastic parameters, the liver damping ratio and splenic shear stiffness measured in 60-Hz 3D MRE were correlated with HVPG. Danielsen et al. (122) assessed whether MRE of the liver or spleen reflected the severity of PH in cirrhotic patients and whether the effect of NSBB on liver and spleen stiffness could be a reflection of its efficacy on portal pressure. The results suggest that liver or spleen stiffness estimated by 2D MRE could reflect the degree of portal hypertension in patients with cirrhosis, but changes in stiffness after NSBB could not predict the effect on HVPG. Besides, Wagner et al. (123) carried out the combination of DCE-MRI and MRE to determine portal hypertension. The results showed that the combination of liver stiffness and perfusion indicators had good accuracy in diagnosing CSPH.

Overall, the measured area of MRE was larger than USE, and MRE could even simultaneously acquire liver stiffness and spleen stiffness (112). However, compared to USE, the research data in MRE were relatively insufficient, and it was not feasible in many countries and regions due to its complicated, time-consuming, and expensive operation. Therefore, this kind of detection may be more suitable for scientific research than clinical practice at the present stage.

### Green cluster 2: The evaluation of portal hypertension-induced variceal bleeding

Green Cluster 2 includes 26 keywords shown in Figure 5D, mainly about “the evaluation of portal hypertension-induced variceal bleeding.” In this situation, the platelet count was important in the risk stratification of PH and the prediction of variceal hemorrhage, and the combination of the platelet count and the spleen diameter was also mentioned.

Esophagogastric variceal bleeding is a common and severe decompensation event of portal hypertension. Esophagogastroduodenoscopy was the gold reference to evaluate varices, and its application was limited by invasiveness and low tolerance of patients. As the hemorrhage risk of varices significantly decreased when HVPG < 12 mmHg, some studies used esophagogastric variceal bleeding as the observation endpoint or standard to evaluate the PH prediction capability of non-invasive methods. In turn, the performance of detecting HVPG ≥ 12 mmHg could also be regarded as predicting variceal bleeding. Hence, most of the non-invasive predictors described above were also studied in evaluating esophagogastric varices, like ICG-r15 could identify patients at high risk for esophageal varices and have similar performance with HVPG (AUROC, 0.859 vs. 0.816) (59); SHAPE could screen high-risk variceal bleeding with AUROC of 0.95 (78); other indices as PLT, AST, ALT, VWF-AG, LSM, and SSM were also discussed (124–127). Among these, the combination of LSM by TE < 20 kPa and the platelet count > 150 G/L was the most recognized surrogate of endoscopic screening, which just missed 0–2% of high-risk varicose veins (40); the platelet count/spleen diameter ratio also received much attention, and the platelet count/spleen diameter ratio > 909 would not miss any esophagogastric varices (128). In the renewing BavenoVII, the patients with LSM of TE ≥ 20 kPa or platelet count ≤ 150 L × 10^9^L needed an endoscopic screen, but the endoscopy could be avoided when SSM of TE ≤ 40 kPa.

## Limitation

This review was based on bibliometric analysis and reviewed the non-invasive methods of PH monitoring according to the keywords clustering results of VOSviewer. The limitations included that the search strategy could not cover all the literature about non-invasive tools on PH. Besides, inequality in the quality of the retrieved literature might influence the credibility of the analysis results. However, the retrieved literature was enough to show the basic architecture of this field and supported further system retrieval.

## Conclusion

**FIGURE 6:**
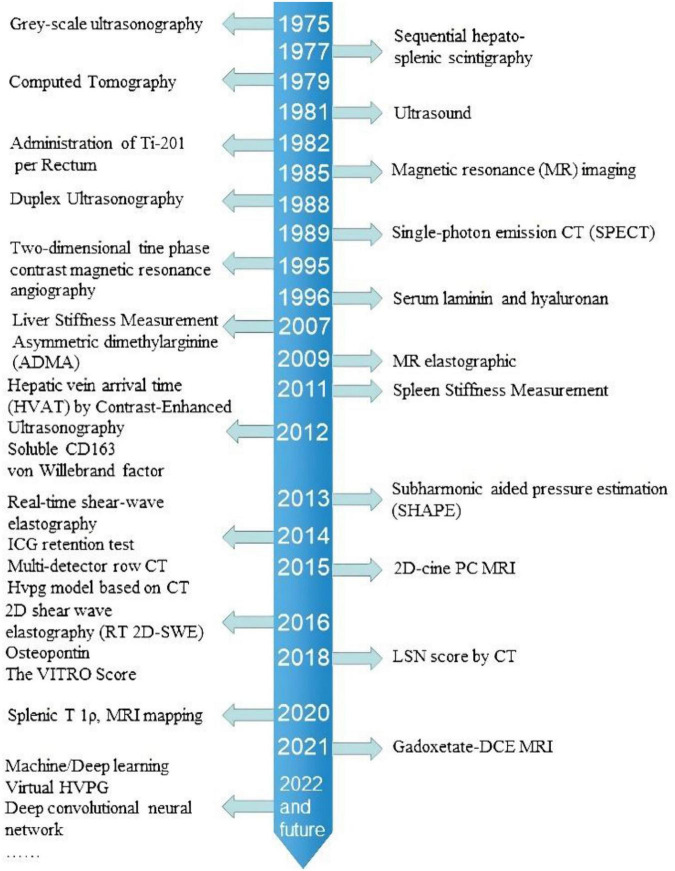
Shows the occurrence time of different non-invasive detection of portal pressure.

The portal vein was away from the surface of the body, making direct puncture a high-risk operation. WHVP was first proposed as an indirect method to measure portal pressure in the 1950s (129), from the 1970s to the 1980s, a large body of studies focused on validating the correlation between WVHP and portal pressure (130). Now, HVPG has been the golden standard to evaluate PH (17). In 2004 (131), a EUS-guided portal vein puncture and pressure measurement succeed in pigs and then succeeded in patients in 2017 (132). EUS-guided pressure measurement of the portal vein and the hepatic vein (or inferior vena cava) obtained a real PPG, which might be useful in diagnosing other types of PH than cirrhosis. However, EUS was still invasive and risky, not suitable to monitor PH continuously throughout the natural course of chronic liver disease. Hence, monitoring PH non-invasively was an inevitable development, and the time axis of the emergence of various non-invasive methods is shown in Figure 6.

As listed in Tables 8, 9, blood tests, US, CT, and MRI provided various features and were studied in different clinical settings. Serum markers often worked as a complement to the LSM, and the new serum markers were continuously appearing with the deepening of the pathophysiological studies of liver fibrosis and portal hypertension. The application of DUS to predict PH was early described in the 1990s, which provided real-time hemodynamic information but was limited by a high-failure rate and inter- or intra-observer variance. The appearance of CEUS in this field was in the 2010s, and SHAPE exploited new ideas on portal hypertension assessment but was still limited by the intrinsic defect of the US. CT and MRI could provide both fibrotic and hemodynamic information about portal hypertension, and MRI has been studied more in this area. The studies focusing on the diagnostic performance of USE on PH were started in the 2000s. LSM of TE was supported by the largest body of literature, and LSM ≥ 25 kPa was widely recognized to diagnose CSPH. The rising of quantitative imaging techniques opened the door to a brand-new world of portal hypertension prediction. The abundant possibilities of imaging analysis were appealing, but the results need rational interpretations.

**TABLE 8:** Different lesions correspond to the examination.

Feature	Methods
Liver fibrosis	USE
Spleen fibrosis	MRE
	Liver surface nodules
	MRI-mapping
	Radiomics
Blood flow of portal system	ICG
	Cholate
	DUS
	CEUS
	MRI with contrast
	CT with contrast
	Computational model
Hepatic function	AST
	ALT
	ALB
	INR
	ALB
Fibrogenesis related molecular mechanism	OPN
	TIMP1
	PIIINP
	HA
	ADMA
	VWF-AG
	CD163
	SCFAs

**TABLE 9:** Screening criteria and prediction models for different types of diseases.

Diseases	Applicable methods	Criteria for prediction
Cirrhosis caused by HCV virus	SSM and LSM by US elastography	HVPG = −4.44 + 0.241*LS + 0.226*SS (*R*^2^ = 0.85, *p*<0.00001).
After the TIPS	SSM by US elastography	SSM was significantly reduced after TIPS surgery.
Hepatitis B cirrhosis	von Willebrand Factor	vWF (1510.5 mU/mL and 1701 ’/mL) had higher positive predictive values for clinically significant and severe portal hypertension (PPV, 90.2 and 87.5%).
Alcoholic cirrhosis	LSM by US elastography	32.2 kPa, for diagnosing HVPG ≥ 10 mmHg (94.5% PPV) and 36.6 kPa, for diagnosing HVPG ≥ 12 mmHg (91.0% PPV).
Alcoholic liver disease, chronic hepatitis B, chronic hepatitis C, and non-obese non-alcoholic steatohepatitis (NASH)	LSM by US elastography	LSM ≥ 25 kpa is considered to be the optimal threshold for determining CSPH.
NASH	Body mass index, LSM, and platelet count	LSM ≤ 15 kPa plus platelets ≥ 150*10^9^/L could rule out CSPH caused by most causes.
NCPH	SSM and LSM by MRE and MRI	LSM of NCPH was significantly lower than that of CPH, SSM/LSM ratio was significantly higher than that of CPH, and LSM < 4.7 kPa could effectively exclude CPH.

LSM, liver stiffness measurement; US, ultrasonic; MRE, Magnetic resonance enhancement; SSM, spleen stiffness measurement; HVAT, hepatic vein arrival time; CEUS, contrast-enhanced ultrasonography; MRI, Magnetic resonance imaging; CT, computerized tomography; MDCT, multi-detector row computed tomography; ICG, Indocyanine green; RHP, regional hepatic perfusion; sCD163, soluble CD163; ADMA, Asymmetric dimethylarginine; HCV, Hepatitis C virus; HVPG, Hepatic Venous Pressure Gradient; Tips, transjugular intrahepatic portosystemic stent-shunt; NCPH, Non-cirrhotic portal hypertension.

As far as detecting changes in portal pressure after various therapies, LSM was unreliable in evaluating the effect of NSBB, and NSBB could not significantly change the stiffness of the liver (122). While hemodynamic factors such as SSM and the portal vein diameter showed a correlation with the changed PH (104, 108, 133). In the situation of antiviral therapy, SSM could also reflect changes in portal hypertension (134). However, as a result of insufficient data, Baveno VII did not recommend using SSM or other non-invasive indices to assess changes in PH (17). Although there is still a gap between all the current non-invasive testing methods and HVPG, we believe that, with further research, non-invasive prediction of portal hypertension will replace or even surpass invasive testing.

## Author contributions

MN and XL contributed to the conception, design, and final approval of the manuscript. JX, SW, AA, LW, and TR contributed to sequencing data. XS and HN wrote the final manuscript. All authors have read and approved the final version of the manuscript.
